# Prevalence of elevated blood lead levels and risk factors among children living in Patna, Bihar, India 2020

**DOI:** 10.1371/journal.pgph.0000743

**Published:** 2022-10-05

**Authors:** M. J. Brown, P. Patel, E. Nash, T. Dikid, C. Blanton, J. E. Forsyth, R. Fontaine, P. Sharma, J. Keith, B. Babu, T. P. Vaisakh, M. J. Azarudeen, B. Riram, A. Shrivastava

**Affiliations:** 1 Department of Social and Behavioral Sciences, Harvard Chan School of Public Health, Boston, Massachusetts, United States of America; 2 National Center for Disease Control India, New Delhi, India; 3 Pure Earth, New York, New York, United States of America; 4 Division of Global Health Protection, Centers for Disease Control and Prevention, Atlanta, Georgia, United States of America; 5 Stanford Woods Institute for the Environment, Stanford University, Stanford, California, United States of America; Health Effects Institute, UNITED STATES

## Abstract

Childhood lead exposure remains a key health concern for officials worldwide, contributing some 600,000 new cases of intellectually disabled children annually. Most children affected by high exposure to lead live in low- and middle-income countries. The leaded gasoline phase out in India was completed in 2000. Yet, in 2020, an estimated 275 million children aged 0 to 9 years had blood lead levels (BLLs) ≥ 5 μg/dL known to adversely affect intelligence and behavior. Lead sources reported in India include spices, cookware, paint, traditional medicines and cosmetics, and lead-acid battery recycling and repair. However, their relative contribution has not been characterized. More than 200 lead pollution sites related to battery recycling and repair activities were identified in Bihar and Jharkhand, India. Ninety percent of the recycling sites had soil lead concentrations exceeding the US Environmental Protection Agency’s standards. We compared blood and environmental lead levels in two groups of children in Patna, Bihar. Households in proximity to battery recycling operations (Proximal n = 67) versus households distal to these operations (Distal n = 68). The average age of children was 40 months; 46% were female. Overall, the geometric mean (GM) BLL was 11.6 μg/dL. GM BLLs of children in Proximal and Distal households were not significantly different (10.2 μg/dL vs. 13.1 μg/dL respectively; p≤0.07). About 87% children, 56 Proximal and 62 Distal had BLLs ≥5 μg/dl. Lead concentrations in environmental samples were significantly higher in Proximal households (soil mean 9.8 vs. 1.6 μg/ft2; dust mean 52.9 vs. 29.9 μg/ft2 p<0.001; Proximal vs. Distal respectively) whereas concentrations in all spices were higher in Distal households (mean 46.8 vs 134.5 ppm p<0.001; Proximal vs. Distal respectively), and turmeric (mean 59.4 vs. 216.9 ppm Proximal vs. Distal respectively). In multivariate analyses for all children lead in spices and turmeric and number of rooms in the house were significant while for the Proximal group only lead in spices remained in the model. The predictive value of these models was poor. For the Distal group, a model with lead concentration in spices, turmeric and soil and number of rooms in the house was a much better fit. Of the 34 water samples collected, 7 were above the Indian standard of 10 ppb for lead in drinking water (2 in the Proximal area, 5 in the Distal area). Children in Patna, Bihar, India are exposed to multiple sources of lead, with lead levels in house dust and loose, locally sourced spices the most likely to increase blood lead levels. A holistic approach to blood lead testing and source identification and remediation are necessary to prevent lead exposure.

## Introduction

Lead is a cumulative neurotoxicant and non-degradable environmental contaminant. It is added to countless consumer products including paint, teas, and cosmetics and to industrial emissions from manufacturing processes [1]. For children less than 6 years old the dominate exposure pathway is lead in settled dust, soil and toys or jewelry ingested during normal hand-to-mouth behavior and when ingested food or water are contaminated by lead. Their higher gastrointestinal absorption rates, and higher rates of iron deficiency enhance lead absorption. Children can also inhale lead dust and fumes and higher relative air intake increases the risk for high blood lead levels (BLLs) [1]. High BLLs are particularly detrimental to children due to their developing central nervous system [1]. According to a recent joint report by Pure Earth and the United Nations Children’s Fund (UNICEF), globally about 0.8 billion children were estimated to have BLLs ≥5 μg/dL, a level at which the World Health Organization (WHO) recommends medical follow up and environmental investigation and remediation [2, 3]. Low and Middle income countries (LMICs) in South Asia and Africa have a greater burden of lead related mortality and morbidity than higher income countries [4]. Lead affects all the organs in the body and has adverse effects on neurological, reproductive, and cardiovascular systems at BLLs well below 5 μg/dL [5]. For children, these include increased risk for neurological damage leading to intellectual and behavioral deficits that persist into adulthood [6]. Lead exposure accounted for 53% of the global burden of idiopathic developmental intellectual disability among children younger than 5 years in 2017 [2]. In children followed from infancy to age 5–10 years, a reduction of 6.9 IQ points is observed BLLs increase from 2.4 to 30 μg/dl, with a steeper decline in intellectual abilities at BLL < 7.5 μg/dl [6]. At a population level, 5 points loss in IQ causes a >50% increase in the proportion of individuals with intellectual disabilities and a >50% decrease in exceptionally intelligent people, impacting societal development and stability [7].

The Institute for Health Metrics and Evaluation found that India experienced nearly 7 million lead-attributable disability adjusted life years (DALYs) and more than 232,500 deaths in 2019 [8]. Although gasoline in India was certified as lead free in 2000, in 2020 an estimated 275 million and 64 million children aged 0 to 9 years had BLLs ≥5 μg/dL and ≥10 μg/dL, respectively [9].

Informal recycling of used lead-acid batteries (ULABs) (e.g., the unregulated, breaking and backyard smelting of used batteries to retrieve lead) has been identified as a highly concentrated source of lead exposure [7]. Lead-acid batteries are used to power vehicles and store alternative energy produced by wind and solar power. As these batteries complete their useful life, which can be as short as 2 years, they are readily recycled in the informal sector, with severe environmental impacts.

In 2016 and 2017, more than 200 suspected lead battery manufacturing and ULAB recycling activities were identified in Bihar and neighboring state of Jharkhand, India [10]. Lead concentrations in soil in > 90% of 84 sites were found to have concentrations exceeding the US Environmental Protection Agency’s standard for lead of 400 ppm in bare soil in play areas and/or 1200 ppm in non-play areas; in 10% of sites lead concentrations > 100,000 ppm were identified [11]. Through the release of lead dust and lead vapor, ULAB recycling can result in soil contamination beyond the work area into adjacent neighborhoods and also contaminate in local food products.

In India, informal ULAB recycling accounts for an estimated 50% of countrywide lead recycling producing 700–750 metric tons of lead [12]. Recycling is expected to increase further with expansion of the economy and demand for electric vehicles including electric rickshaws.

In addition to lead exposure relating to ULAB recycling operations, there are a number of other potential lead exposure sources including adulterated spices, lead contaminated aluminum cookware, paint, traditional medicines, cosmetics, and other environmental impacts. A study conducted in Mumbai and Delhi 1998–1999 found that age ≥12 months, low standard of living and ≥ 95% height/weight vs. < 5% predicted high BLLs. However, this study was conducted before gasoline in India was certified as lead free and gasoline contribution the children’s BLLs was not measured [13]. Similarly, a later study postulated that defective housepaint was a major source of lead for children in Aligarth but the lead content of the paint was not measured [14]. The relative contributions of various sources of lead have not previously been characterized within India and there is little data available characterizing exposure to lead from ULAB recycling versus other potential lead sources and children’s BLLs.

We measured BLLs and assessed risk factors and the various sources of lead exposure in children in 2 study groups in Bihar. We hypothesized that children living close to ULAB operations (Proximal households) would have higher blood and environmental lead levels than children living further away (Distal households), as detailed below. Results of the study offers insight into baseline BLLs among children and the sources of such lead exposure in households of Patna, Bihar.

## Methods

We used a modified two-stage sampling approach based on the Community Assessment for Public Health Emergency Response (CASPER) surveys has been widely field tested [15].

### Stage one

The first stage of cluster sampling involved defining the geographic population of interest and dividing that population into mutually exclusive units to form the cluster sampling frame. From the sample frame, each cluster was defined, enumerated and then selected. Cluster selection is probabilistic and selected clusters were mapped and listed.

### Stage two

In the second stage households were systematically selected in each of the clusters. The cluster is the primary sampling unit, and the household is the secondary sampling unit. A sample of households from the stage two sampling frame was selected from within each cluster by random selection. Once the desired number of households were achieved within each cluster, households were systematically visited until children 9–71 months of age are identified and enrolled [16].

### Study site

We enrolled 135 children < 6 years old. In the Proximal group were 67 children living in one of two lead pollution hotspots located near a point source related to ULAB activities within residential areas in Patna, Bihar. ULAB activities were previously documented through Pure Earth’s Toxic Sites Identification Program (TSIP), using local investigators trained to identify and assess contaminated sites with a rapid assessment screening protocol. The Proximal households were those with children <72 months old (n-67), selected randomly using geocoordinates from within 0.3 km radius of the ULAB sites. The 0.3 km radius to definition of exposure is based on estimates that about 50% of airborne lead at ULAB sites is settled within that boundary. A Distal group of 68 children were randomly selected from sites located more than 5.8 km radius from the boundaries of the hotspots and visually inspected by trained staff to ensure that there were no ULAB activities underway in these areas. The 5.8 km radius definition is well above the average distance from a point source where elevated blood lead measures have been reported based on findings from studies measuring blood lead levels and point source exposures [17–19].

### Participant selection

We selected the residential building nearest to a randomized set of geocoordinates. If no household with eligible children was found within the first two levels of the building, the second house to the right and then the left was approached. Thus, up to three eligible households were searched in different directions using one set of geocoordinates. One child <72 months old was selected from each household. A younger child, ≥ 6 months old, was selected preferentially if there were multiple eligible children. After the child’s parent or guardian consented to participate, native Hindi speakers collected demographic and child’s health and behavioral data using an oral semi-structured survey. The interview data were collected digitally in Epi Info 7. Venous blood samples from participant children were collected by trained pediatric phlebotomists.

### Environmental sample collection

Human exposure pathways were evaluated including water, soil and food by trained Epidemic Intelligence Service Officers supervised by experienced lead risk assessors.

Dust samples were collected from each household according to the US CDC protocol [15]. An average of three dust samples were taken from each household, including a floor and a windowsill where present. Of the 339 dust samples, 49 (15%) of families, such as those living outside or with earthen floors and no windows, a hard furniture surface was sampled. Samples were collected with Ghost Wipes^®^ nonwoven polyvinyl alcohol fiber wipes moistened with deionized water (ASTM E1728). Wipes were sealed in polyethylene bags and analyzed by Environmental Hazard Services LLC (Richmond, VA, USA) a laboratory certified for environmental analyses using flame AA instrumentation using EPA method SW846/7000B.

Lead levels in ground spices, painted walls and doors, plastic items, cosmetics (dry), food items (powder form), cookware, and surrounding soil were measured and recorded on-site using either of 2 portable X-ray fluorescence (pXRF) machines–Olympus Delta DCC-4000 (equipped with soil and paint modes) and Niton XL3t 700S GOLDD (equipped with soil, paint, metals, and plastic modes). The pXRF was adjusted to the appropriate mode for each media.

The use of the pXRF as a screening tool has been validated for soil [20, 21], spices [22, 23]; plastics [23]; cosmetics and dietary supplements [24] and paint [25]. There is currently no standard method for lead analysis in metal cookware; however, the use of the pXRF for screening metal content in aluminum cookware has previously been demonstrated [26].

The calibration was field checked against a standard with a known concentration. For quality control, duplicate soil samples from 5% of households and analyzed by Black Globe Ecocare Pvt. Ltd (Delhi, India) via atomic absorption spectrophotometry. The lab analysis of these 7 soil samples returned consistently lower results than the pXRF. This could result from heterogeneity within the collected sample. pXRF readings are limited to a very thin layer at the top the soil (approximately 2mm). Although investigators were trained to scrape soil during sample collection, these samples would inevitably include soil from a greater depth. Because we suspect aerial deposition of lead in Patna, lower depths of soil may contain lower concentrations of lead. Furthermore, at many houses, the areas outside were paved, although dusty. pXRF samples were taken in cracks or corners where dust and dirt accumulated. Samples were taken for laboratory analysis by scraping the dust into a bag and shipping it to the laboratory testing. This method, though necessary due to field conditions, is a likely cause of differences between the field pXRF and laboratory results—the soil in cracks and corners likely has laid there for some time, with Pb deposition on top, while the scraping process to get a baggable sample necessarily was from a wider and likely deeper area. Other possible factors for the discrepancy between the pXRF results and the lab are the moisture content of the sample and incomplete digestion of the soil sample by the lab. Nonetheless, the pXRF measures most likely represent the exposure to children though normal hand to mouth activity.

Laboratory analysis of other media assessed with the pXRF was not possible due to budgetary and logistical constraints.

### Spices

Spice samples with lead concentrations > 100 ppm were collected for possible laboratory testing with permission of the parent/guardian. The sampling effort focused primarily on spices of a yellow, orange or red color, as these colors can be achieved naturally or enhanced via adulteration with lead chromate or lead oxide.

A total of 17 spice samples were selected among those with the highest lead concentrations (>1,000 ppm for turmeric and >100 ppm for chili and coriander) as measured by pXRF, in order to conduct follow-up laboratory measurements at Stanford University’s Environmental Measurements Facility (https://emf1.stanford.edu/). The lead concentrations determined by ICP-MS for this subset of spices samples was highly correlated with the results from the pXRF (R^2^ = 0.9789).

The follow-up measurements ascertained the most probable pigment type used to adulterate the spices by comparing the lead and chromium concentrations, as well as the lead speciation of samples with those of known pigments. Samples were analyzed in duplicate to determine lead and chromium concentrations and the molar ratio of lead:chromium via Inductively-Coupled Plasma Mass Spectrometry (Thermo Scientific XSERIES 2 ICP-MS) at Stanford’s Environmental Measurements Facility (em1.stanford.edu). Powdered spice samples were dissolved in concentrated 69% nitric acid and digested via microwave digestion (MARSXpress, CEM Corporation). Root samples were soaked in 35% nitric acid to remove the powder coating then digested via microwave digestion. Samples were further diluted to 2% nitric acid for analysis via ICP-MS. An internal standard solution and blanks were analyzed at least every 30 samples for quality control. Additional confirmatory analyses were conducted via X-Ray Diffraction (XRD, Rigaku mini flex 600) using a copper source and silicon strip detector to determine the dominant lead species (e.g., lead chromate, lead oxide, and lead carbonate).

### Water

Thirty-four representative water samples were collected to characterize the primary drinking water sources in each target neighborhood; borewells, dug wells, and city distribution pipes. The water samples were collected from the primary drinking water source at the time of the risk assessment. Samples were collected in acid-washed 250 mL bottles suitable for metals analysis. Once collected, 24 of the samples were treated with nitric acid to reduce the pH to ~2 and refrigerated. Water samples were analyzed by Black Globe, via method IS 3025(P-47)-1994.

### Comparison to regulatory standards

We calculated the number and percent of environmental samples that were above existing regulatory standards using Indian standards when available or if not using US standards. The Central Pollution Control Board of India (CPCB) has adopted ≤ 90ppm as the standard for lead in new paint. A standard for previously painted surfaces is not available. We have adopted the US Housing and Urban Development (HUD) standard for painted residential surfaces of 1 mg/cm^2^ to approximate 90 ppm [25].

### Blood lead determination

Venous BLLs (n = 135) were collected, refrigerated, and analyzed within 24 hours of sample collection by one analyst (MJB) using a point of care (POC) instrument with screen printed electrode technologies based on anodic stripping voltammetry. The POC instrument has a reportable range of 3.3–65 μg/dl. Kit controls and duplicates were run every 20 samples and with every change in test kit batch consistent with US Clinical Laboratory Improvement Act regulations. Blood lead kit controls were composed of lead salt in buffered aqueous solution with bovine serum albumin. Two levels of quality control material are provided with the test kit, designated “Level 1” and “Level 2”. The instrument was recalibrated with every batch.

Only venous blood was collected. In areas of high ambient lead contamination, obtaining a clean capillary blood may not be feasible and follow up of elevated capillary BLLs may be time consuming and result in an unacceptable loss to further care by a health care provider if parents are unable to access confirmatory venous blood lead levels. However, analyzing venous BLLs on currently available POC instruments is an off-label use. Some venous blood tubes may provide falsely low BLL test results because the compound thiuram is used in the rubber caps and causes an interference. For this study, we used venous blood collection tubes without thiuram.

### Data analysis

Descriptive analysis included household and child characteristics. The data was analyzed using SAS version 9.4. Due to the right skewness of the lead values, BLLs and environmental lead levels were transformed using the natural log. We compared geometric mean BLLs, household characteristics, distribution of environmental lead levels in households of Proximal and Distal populations using a Chi^2^ for categorical variables and a t-test for continuous variables or, when necessary, Fisher’s exact test. For BLLs below the limit of detection of 3.3 μg/dL, the actual value reported by the instrument was used in the data analysis to avoid introducing a positive bias. Environmental lead levels samples were recorded as zero for those families who live outside in informal settings (n = 2). Zero values and those < detection limit were replaced with imputed values by dividing lowest detected positive value in the sample category by the square root of two [27]. One observation with very high lead concentration in paint was removed based on Tukey’s criteria. Multiple results within each of the environmental media category were averaged to estimate a single lead exposure level per sample category per household. Spices were analyzed separately as turmeric versus other spices as turmeric has been found to have high lead concentration when adulterated by yellow pigment [28]. The ratio of lead levels in environmental samples were compared between Proximal and Distal groups. Mean environmental lead levels were also compared to available regulatory standards.

We used linear regression to examine the relationship between risk factors, demographics and tercile of lead concentration in environmental media all children (Combined) and stratified by distance to ULAB activities (Proximal vs. Distal). Bivariate analyses were conducted to assess association of each risk factor with elevated BLLs. The risk factors assessed included age, gender, hand-mouth behavior, use of traditional medicine, use of the traditional eye cosmetic kohl, family characteristics, physical characteristics of the household, involvement of family members in ULAB and other lead related activities (painting, renovating), presence of ULAB related items in house, smoking in family, recent renovation and, or painting in house, and diet demographic data included child’s age and sex, number of rooms in the house and type of flooring.

Those variables found significant in bi-variate analyses at p-value ≤0.1 were included in multiple linear regression analyses. Backward regression method was used to build full and parsimonious models. We did a stratified analysis We constructed regression models separately for all enrolled children and then a stratified analysis for by exposure status. Figures, stratified by group, were constructed using R which includes outliers in calculations, but outliers were removed from the graph to enhance visibility.

We used principal component analysis to explore the relationship between lead concentrations in environmental samples and those in spice samples.

At the end of the study, blood and environmental lead results were shared with 134 of the 135 families, and health education was provided if BLLs ≥ 5 μg/dL or high environmental samples were found. This included an explanation of the environmental samples and ways to limit children’s exposure to specific sources of lead and for those children with high BLLs recommendations that families follow up for additional blood lead testing with their pediatric health care provider. At least 2 attempts were made to return results to the remaining participant. However, the onset of the COVID-19 pandemic in March 2020 prevented further in-person follow-up. These restrictions also halted more extensive health education and referral efforts for children with BLLs ≥30 μg/dL.

### Human subject review

This study was approved by the Ethics Committee of the Indian Council of Medical Research (ICMR)–Rajendra Memorial Research Institute of Medical Sciences (REF: 14/RMRI/EC/2019). The approved consent form was read to parents or guardians of children before enrollment by a native Hindi speaker after parents signed or marked the form, they were provided with a copy. Financial support was provided by the United States Agency for International Development (USAID). The opinions expressed herein are those of the authors and do not necessarily reflect the views of the study sponsors. The funders had no role in study design, data collection, analysis, or data interpretation.

## Results

### Demographics

We enrolled 135 households, 67 from residential areas proximal to ULAB recycling sites and 68 from areas without these sites. Average age of the children enrolled was 40 months (range = 8–71 months, sd = 15.9 months). Sixty two (46%) were female (Table 1).

**Table 1:** Participant characteristics and risk factor distribution for all children and stratified by distance (Proximal vs. Distal) to ULAB activities. Patna, Bihar, February 2020.

Participant demographic and risk factors	Combined		Proximal		Distal		*p-value* Proximal vs Distal
	n = 135		n = 67		n = 68		
	n	%	N	%	n	%	
**Participant child demographics**							
8–23 months	25	19	14	21	11	16	0.06
24–47 months	64	47	25	37	39	57	
48–71 months	46	34	28	42	18	26	
Male	73	54	39	58	34	50	0.34
**Family demographics**							
1 to 4 Adults	82	61	46	69	36	53	.06
>4 Adults	53	39	21	31	32	47	
≤2 children	111	82	54	81	57	84	0.62
>2 children	24	18	13	19	11	16	
Pregnant woman living in house	13	10	6	9	7	10	0.79
**Household demographics**							
House type							
Apartment	20	15	15	22	5	7	<0.01
Individual	113	84	50	75	63	93	
Living Outside	2	2	2	3	0	0	
**Level of household in the building**							
Ground	110	82	52	78	58	85	0.49
1^st^ floor	14	11	8	12	6	9	
> = 2^nd^ floor	11	8	7	10	4	6	
**Floor type**							
Dirt	43	32	19	28	24	35	0.39
Concrete	92	68	48	72	44	66	
**No of rooms**							
≤3	94	70	51	76	43	63	0.11
>3	41	30	16	24	25	37	
**Family lead exposure**							
Used Lead-battery (ULAB) exposure							
Any family member in ULAB activity	8	6	6	9	2	3	0.16
In-house operation	1	1	0	0	1	1	1.0
Material stored around house*	19	14	11	16	8	12	0.44
Outside house	7	37	4	36	3	38	1.0
Inside house	12	63	7	64	5	63	
**Occupational Lead exposure of any family member in past 6 months**							
None	125	91	62	93	63	93	1.0
One	7	5	4	6	3	4	
Multiple^#^	3	2	1	2	2	3	
**Lead exposure due to activities in and around house in past 6 months**							
None	97	72	45	67	52	76	0.46
One removing paint/renovating	25	19	14	21	11	16	
Multiple^†^	13	10	8	12	5	7	
**Spices used in past week** (Y/N)							
Turmeric	135	100	67	100	68	100	-
Chili powder	131	97	66	99	65	96	0.62
Mixed spices	126	93	63	94	63	93	1.00
Ginger	126	93	61	91	65	96	0.33
Cumin powder	126	93	59	88	67	97	0.02
Cinnamon	114	84	58	87	56	83	0.50
Cardamom	114	84	57	85	57	84	0.84
Clove	105	78	51	76	54	80	0.64
Coriander powder	98	73	48	72	50	74	0.81
Fenugreek	67	50	35	52	32	48	0.55
**Spice purchase**							
Loose supply	106	79	43	64	64	93	<0.01
National brands	32	24	24	36	8	12	<0.01
Locally packed brands	6	4	5	8	1	1	0.11
**Primary drinking water source**							
Treated tap^† †^	64	47	39	58	25	37	0.01
Untreated borewell	71	53	28	42	43	63	
**Use of utensils**							
Metal cookware	135	100	67	100	68	100	
Ceramic dishes	5	4	3	5	2	3	0.68
Metal containers	72	53	40	60	32	47	0.14
Plastic containers	74	55	34	51	40	59	0.35
**Smoker in household**	62	46	27	40	35	51	0.19
**Participant child food consumption and behavior**							
Meat consumption	112	83	54	81	58	85	0.47
Fish	108	80	52	78	56	82	0.49
Mutton	104	77	52	78	52	77	0.87
Egg consumption	120	89	58	87	62	91	0.39
Puts toy/paint chip/object/dirt in mouth							
Less than once a week/never	33	24.81	15	22	18	26	0.81
Several times a week	26	19.55	14	21	12	18	
Several times a day or more	74	55.64	36	54	38	56	
Child’s main play area							
In and around the house	102	76	46	69	56	82	0.15
Outside the house	16	12	11	16	5	7	
School	17	13	10	15	7	10	
Child wears	27	20	11	16	16	24	0.30
Cosmetic use, kohl	43	32	20	30	23	34	0.62
**Blood lead level μg/dL**							
**Geometric Mean (STD)**	135	12 (2.2)	67	10 (2.3)	68	13 (2.1)	0.07
**Median**		13		13		14	
≥5 **μg/dL**	118	87	56	84	62	91	0.18
≥10 **μg/dL**	92	68	41	61	51	75	0.09

* p≤ 0.0 5μg/dL

Compares the distribution of factors known to predict blood lead levels (BLLs) in children for children living in neighborhoods with ULAB processing sites (Proximal n = 67) vs. children living in neighborhoods without these sited (Distal n = 68) and Overall (n = 135).

Proximal children were more likely to live in apartments, compared to Distal children. Distal children were more likely to use cumin and locally sourced loose spices, drink water from bore hole wells (deep artisanal wells) and be 2–4 years old. The geometric mean BLL was higher in Distal children, but the difference did not reach statistical significance (p> 0.05).

Of the 15 demographic, household and environmental factors measured, 3 were significantly different by group (Table 1). However, for most of these variables the groups were similar. Proximal group children were older and lived in homes with ≤ 3 rooms but these differences were not statistically significant. The groups were also similar in the number of households living on the ground floor and with concrete vs. dirt floors. There were no significant differences in children’s hand-to-mouth behavior, play area, metal jewelry wearing or kohl use (Table 1).

### Blood lead

The arithmetic mean BLL for all study participants was 15 μg/dL (range = 0.8–53.9 μg/dl, sd = 9.8) and geometric mean (GM) was 12 μg/dL. The GM BLLs of children in Proximal and Distal households were 10 μg/dL and 13 μg/dL respectively (p≤0.07). About 87% children, 56 (84%) in the Distal group and 62 (91%) in the Proximal group had BLLs ≥5 μg/dl (Table 1).

### ULAB

In most ULAB sites the dominant pathway for children, settled lead contaminated house dust and soil from smelting emissions, produces the majority of human health risks [29]. However, there were no significant differences between the groups in the numbers of households with a family member engaged in ULAB activities, in-house ULAB operations, and ULAB material or battery storage around/in the house. Having a family member working in lead-related occupations other than ULAB and in-house construction or repair activities were also not significantly different in Proximal vs. Distal households (Table 1).

### Spices

Spice usage in the past week was similar across the groups except that Distal group households were more likely to use cumin powder (97% vs. 88% respectively; p = 0.02). There were significant differences in sources of spices between the two groups. Proximal group households were more likely to purchase nationally known brands of spices compared the Distal group (n = 24; 36% vs n = 8; 12%, respectively; p ≤ 0.01). Distal households were more likely to use loose spices purchased at local markets compared to Proximal household (n = 64; 93% vs. n = 43; 64% respectively, p ≤ 0.01) (Table 1).

**Fig 1:**
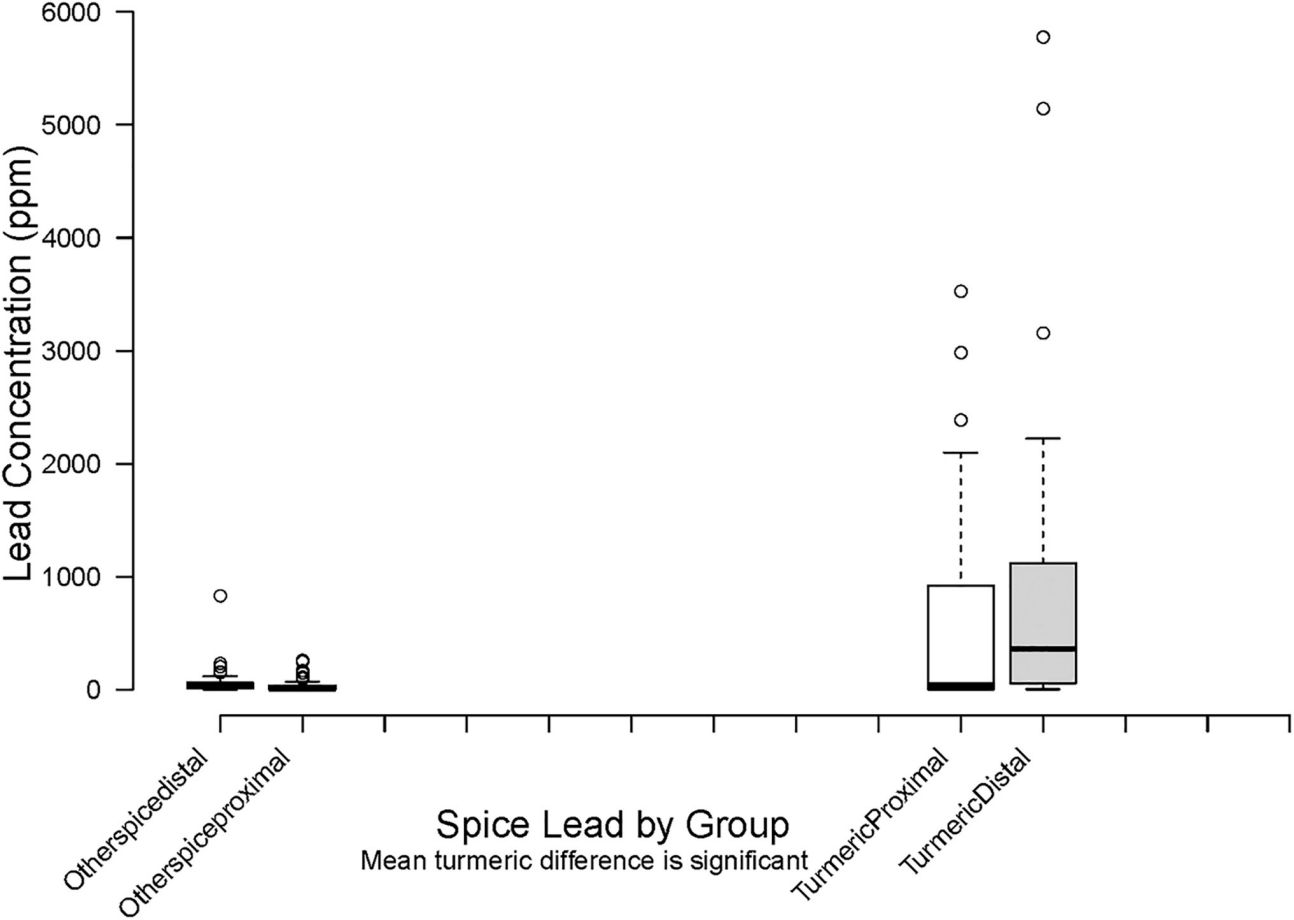
Lead in spices.

In Distal households, lead in turmeric and spices other than turmeric was significantly higher than in Proximal households (n = 52 vs. 41 for turmeric and n = 30 vs. 13 for other spices, respectively, p<0.01) (Fig 1).

Of the 17 samples (10 turmeric, 4 chili and 3 coriander) selected for in-depth laboratory assessments via ICP-MS and XRD, all contained lead and chromium. The average molar lead:chromium concentration was 1.1 for turmeric, 1.2 for chili and 1.3 for coriander. XRD analyses identified lead chromate, but the quantification of complete lead speciation was not possible.

### Water

Significantly more Proximal group households relied on municipality-supplied tap water as primary drinking water source than in the Distal group (n = 39; 58% vs n = 25; 37% respectively) (Table 1). Distal group households were more likely to rely on bore hole wells for potable water. Of the 34 water samples tested most lead levels were below the limit of detection and 7 (21%) were above the US EPA water action level of 15 ppb ranging from 130 to 474 ppb). All 7 water samples with lead levels exceeding the US EPA water action level were drawn from boreholes; 2 were in the Proximal area and 5 were in the Distal area. (Data not shown).

### Food/Cookware

Use of metal cookware and food storage containers was similar across the groups. Food consumption patterns (meat, fish, egg) were similar in enrolled children in both groups. A total of 59 cookware implements were tested including woks, pressure cookers and pots. The lead concentrations in the cookware ranged from non-detectable to 8770 ppm (average 1832.5 ppm). (Data not shown).

### Dust/Soil

Lead concentrations in environmental samples showed wide variation, overall (Table 2). However, lead in dust wipe, soil and paint samples was significantly higher in the Proximal households. Most notably, among the Proximal group, 21 (31%) households had dust lead levels above the US EPA threshold of 40 μg/ft^2^ while in the Distal group there were only 6 (9%) households above the threshold (Table 3).

**Table 2:** Lead levels in household environmental samples for all enrolled children, Patna, Bihar, February 2020.

Environmental sample**	Combined (m = 135)			
			Geometric	
	n	Median	Mean	Sd
Dust wipe (μg/ft^2^)	135	9.5	4.0	22.4
Dust Wipe Concrete Floor (μg/ft^2^)	43	8.3	13.2	5.8
Dust Wipe Dirt Floor (μg/ft^2^)	92	24.1	17.3	9.2
Paint (μg/cm^2^)**	135	0.2	0.7	13.2
Soil (ppm)	130	31.3	40.1	2.4
Spice (ppm)	131	88.7	80.3	7.1
Turmeric (ppm)	128	144.5	114.6	11.0
Other Spice (ppm)	115	19.0	19.3	4.1

* Values for zero tests were imputed.

**Paint was measured in μg/cm^2^ by pXRF

Lead concentrations in environmental samples show wide variation, overall.

**Table 3:** Lead levels in household environmental samples stratified by distance to ULAB activities, Patna, Bihar, February 2020.

Environmental sample	Proximal (n = 67)				Distal (n = 68)				Ratio of Geometric Means Proximal or Distal	*p*-value*
			Geometric				Geometric			
	N	Median	Mean	Sd	n	Median	Mean	sd		
Dust wipe (μg/ft^2^)	67	17.1	9.8	18.1	68	4.8	1.6	21.7	6.0	<0.01
Dust Wipe Floor Concrete (μg/ft^2^)	37	12.7	20.1	7.1	29	4.1	7.7	3.7	2.6	0.02
Dust Wipe Floor Dirt (μg/ft^2^)	14	39.5	58.4	3.7	18	6.9	6.7	10.4	8.7	<0.01
Paint (μg/cm^2^)	67	0.15	1.3	19.6	68	0.15	0.4	7.4	3.3	<0.01
Soil (ppm)	67	41	52.9	2.7	63	35.3	29.9	1.9	1.8	<0.01
Spice (ppm)	64	35.3	46.8	8.1	67	141	134.5	5.4	0.4	<0.01
Turmeric (ppm)	63	44.0	59.4	12.3	65	363	216.9	8.3	0.3	<0.01
Other Spice (ppm)	51	9	11.4	4.5	64	32.5	29.4	3.3	0.4	<0.01

*p-value represents significance level for distribution of mean lead levels in the samples in vs. Distal(t-test)

Dust wipe, soil and paint lead samples were significantly higher in the households of the Proximal children compared to those of Distal children. For the Distal children, lead levels in all spices, spices other than turmeric and turmeric alone were all significantly higher compared to Proximal children.

### Type of floor

The groups did not differ in the number of households with concrete vs. dirt floor (p = 0.39) (Table 1). In analysis for all children and stratified by group GM mean BLL did not differ significantly by floor type, independent of other exposures (Tables 4 & 5).

**Table 4:** Association between BLLs and various risk factors for all children, Patna, Bihar, February 2020.

Characteristics	Combined (n = 135)				
	n	Geometric mean BLL	sd	Ratio of geometric means *(β)*	p-value*
**No of adults in household**					
>4 members	53	9.8	2.2	(ref)	-
= <4 members	82	12.9	2.2	1.3	0.03
**Level of household in the building**					
Ground level	110	12.5	2.1	(ref)	-
First floor	14	10.0	3.1	0.8	0.31
Second/third floor	11	6.4	1.9	0.5	0.01
**Floor type**					
Concrete	92	10.8	2.3	(ref)	-
Dirt	43	13.6	2.0	1.3	0.12
**No. of rooms in house**					
≤3	94	12.8	2.1	1.4	0.02
>3	16	7.5	2.4	(ref)	-
**ULAB material/battery in house**					
Absent	116	12.2	2.2	(ref)	-
Present	19	8.6	2.5	0.7	0.12
**Location of battery in/around household**					
Outside	7	13.5	3.1	(ref)	-
Inside	12	7.3	2.3	0.7	0.12
**No. of lead-related occupational activities family members involved in past 6 months**					
None	125	12.0	2.1	(ref)	-
Any	10	7.7	2.8	0.6	0.09
**No. of lead-related activities conducted in household**					
None	97	12.1	2.0	(ref)	
Any	38	10.4	2.8	0.9	0.31
**Spice purchase source**					
Branded (national) ONLY	24	7.7	2.5	(ref)	-
Loose (local) ONLY	100	12.7	2.0	1.7	0.01
Other Comb on none	11	12.7	3.0	1.7	0.08
**Frequency of kohl use**					
Never	24	7.7	2.5	(ref)	-
Daily	100	12.7	2.0	1.1	0.52
A few times a week/month	11	12.7	3.0	1.1	0.79
**Child bites nails**					
Less than once a week/never	18	11.9	2.3	(ref)	-
Several times a week	21	10.1	2.2	0.8	0.52
Several times a day or more	95	12.0	2.2	1.0	0.96

Children who lived in homes with 4 or more adults, on the second or higher level floor, with less than 3 rooms, ULAB material present inside, spice source or a family member with a lead-related occupation were significantly more likely to have a higher BLL compared to children who did not live in homes with these characteristics. There was no significant difference by floor type, battery materials outside, kohl use, nail biting or number of lead related activities.

**Table 5:** Association between BLLs and various risk factors stratified by group (Proximal vs. Distal), Patna, Bihar, February 2020.

Characteristics	Proximal (n = 67)					Distal (n = 68)				
	N	Geometric mean BLL	sd	Ratio of geometric means *(β)*	Within group p-value*	n	Geometric mean BLL	sd	Ratio of geometric means *(β)*	Within group p-value *
**No of adults in household**										
>4 members	21	7.3	2.2	(ref)	-	32	12.0	2.1	(ref)	-
≥4 members	46	12.0	2.3	1.6	0.03	36	14.2	2.0	1.2	0.34
**Level of household in the building**										
Ground level (ref)	52	11.6	2.2	(ref)	-	58	13.4	2.0	(ref)	-
First floor	8	8.0	3.2	0.5	0.23	6	13.6	3.1	1.0	0.96
Second/third floor	7	5.3	2.0	0.7	0.02	4	9.1	1.3	0.7	0.31
**Type of Floor**										
Concrete	48	10.0	2.4	(ref)	-	44	12.0	2	(ref)	-
Dirt	19	10.9	2.4	1.1	0.71	24	16	2	1.4	0.09
**No of rooms in house**										
≤3	51	11.3	2.3	1.5	0.10	43	15.0	1.8	1.4	0.05*
>3	16	7.5	2.4	(ref)		25	10.4	2.4	(ref)	-
**ULAB material/battery in house**										
Absent	56	10.9	2.2	(ref)	-	60	13.5	2.1	(ref)	-
Present	11	7.4	2.9	0.7	0.17	8	10.7	2.0	0.8	0.40
**Location of battery in/around household**										
Outside	7	6.9	2.7	(ref)	-	5	8.1	1.8	(ref)	-
Inside	4	11.3	4.4	1.7	0.52	3	17.0	1.9	2.1	0.14
**No of lead-related occupational activities family members involved in past 6 months**										
None	62	10.6	2.3	1.0	-	63	13.5	2.0	1.0	-
Any	5	6.7	3.2	0.6	0.25	5	8.8	9.9	0.7	0.21
**No of lead-related activities conducted in household**										
None	45	10.2	2.1	(ref)	-	52	14.1	1.9	(ref	-
Any	22	10.4	2.9	1.0	0.92	16	10.3	2.6	0.7	0.13
**Spice purchase source**										
Branded (national) ONLY	20	8.0	2.5	(ref)	-	4	6.3	2.6	(ref)	-
Loose (local) ONLY	42	11.9	2.2	1.5	0.08	58	13.3	1.9	2.1	0.05*
Other Comb on none	5	7.9	3.3	1.0	0.99	6	18.8	2.9	3.0	0.02*
**Frequency of kohl use**										
Never	47	9.8	2.3	(ref)	-	45	13.1	2.0	(ref)	-
Daily	14	10.1	2.4	1.0	0.88	9	18.1	1.8	1.4	0.23
A few times a week/month	6	15.2	2.6	1.6	0.24	14	10.7	2.3	0.8	0.36
**Child bites nail**										
Less than once a week/never	12	11.7	2.2	(ref)	-	6	12.3	2.8	(ref)	-
Several times a week	11	10.1	2.4	0.9	0.69	10	10.0	2.1	0.8	0.58
Several times a day or more	44	9.9	2.4	0.8	0.55	51	14.3	2.0	1.2	0.63

Among Proximal group children lower mean BLLs were significantly associated with ≤ 4 adult members in a household and living on a second or higher-level floor. For Distal children a higher mean BLL was associated with households that purchased loose spices from local stores compared to those using nationally known brands of packed spices.

### Paint

Of the 135 households, 82 (61%) had measurements ≤ 0.15 μg/cm^2^ (Range 0–2330 μg/cm^2^) (Table 2). Of 72 interior paint samples tested, 3 (4%) were above the 1 mg/cm^2^ lead concentration defining lead hazard (Table 6).

**Table 6:** Comparison of lead levels in environmental samples with regulatory standards, Patna, Bihar, February 2020.

Environmental Sample	No of household tested (N = 136)	Range of lead levels	Household exceeding regulatory lead level n, (%)	Elevated Lead Level Definition
**Soil (ppm)**	**131**	**8.5–3755**	**3 (2.3)**	**400ppm (EPA, USA)**
**Dust floor composite (μg/ft** ^**2**^ **)**	**125**	**0–745.1**	**26 (20.8)**	**40μg/ft**^**2**^ **(EPA, USA)**
**Dust window (μg/ft** ^**2**^ **)**	**22**	**0–73.5**	**0 (0)**	**250μg/ft**^**2**^ **(EPA, USA)**
**Interior Paint (mg/cm** ^**2**^ **)**	**72**	**0–687.5**	**3 (4)**	**1mg/cm**^**2**^ (HUD USA)*
**Spice (ppm)**	**132**	**3.5–2574**	**106 (80.3)**	**10ppm (FSSAI, India)**

We found 106 (80%) households using spices with higher lead levels than acceptable for most spices by Indian standards. Indian standards for soil, and dust were not available. Compared to US Environmental Protection Agency (EPA) standards, elevated lead in dust floor samples were found in 27 (22%) households.

* The Central Pollution Control Board of India (CPCB) has adopted ≤ 90ppm as the standard for lead in new paint. A standard for previously painted surfaces is not available. We have adopted the US Housing and Urban Development (HUD) standard for painted residential surfaces of 1 mg/cm^2^ which approximates.

### Comparison to regulatory standards

As described in Table 6, most spice samples were above the Food Safety and Standards Association of India (FSSAI) regulatory threshold for lead in food of 10 ppm [30]. A total of 26 (21%) of dust floor composites were above the US PA definition of elevated of 40 μg/ft2. Of the 72 paint samples tested, 3 were at or above the lead concentration defined as a hazard by US HUD. No international or Indian standard for XRF lead measurements in cookware are available.

### Increased blood and environmental lead levels

A 10% increase in lead concentration in environmental media was associated with a modest but statistically significant increase in BLLs except for paint and soil for all children and by group. Each 10% increase in dust wipe was associated with 0.7% (Proximal group, p-value = 0.05) and 0.8% (Distal group, p-value = 0.01) increase in mean BLLs, respectively). For turmeric, each unit 10% increase in lead concentration was associated with 0.8% in BLLs (Proximal, p-value = 0.05) and 0.6% (Distal, p-value = 0.11). For the Proximal group a 10% increase in soil lead was associated 1.8% increase in BLL. Thus, a child in the Proximal group whose BLL was the GM for the group of 10 μg/dL, 1.8 μg/dL (p = 0.07) was contributed by soil. (Tables 7 & 8).

**Table 7:** Percent increase in BLL for every 10% increase in environmental lead levels in household samples for all children. Patna, Bihar February 2020.

Environmental	Combined (n = 135)			
Sample Type	n	%Increase in BLL per 10% increase in lead concentration	se	P value
Dust Wipe (μg/ft^2^)	135	0.6%	0.23%	0.02
Paint (mg/cm^2^)	135	-0.05%	0.3%	0.86
All Spice (ppm)	132	1.0%	0.3%	<0.01
Other Spice	116	1.9%	0.5%	<0.01
Turmeric	127	0.8%	0.3%	<0.01
Soil (ppm)	131	0.6%	0.8%	0.40

For all children each 10% increase in lead concentration in dust, all spices, other spices and turmeric was associated with a significant increase in BLL. Soil and paint lead levels were not associated with a 10% increase in BLL.

**Table 8:** Percent increase in BLL for every 10% increase in environmental lead levels in household samples stratified by distance to ULAB activities. Patna, Bihar February 2020.

Environmental	Proximal (n = 67)				Distal (n = 68)			
Sample Type	N	%Increase in BLL per 10% increase in lead concentration	se	Within group p value	N	%Increase in BLL per 10% increase in lead concentration	se	Within group p value
Dust Wipe (μg/ft^2^)	67	0.7%	0.3%	0.05	68	0.8%	0.3%	<0.01
Paint (μg/cm^2^)	67	0.3%	0.3%	0.35	68	-0.5%	0.4%	0.22
All Spice (ppm)	64	1.1%	0.5%	0.02	67	0.5%	0.6%	0.33
Other Spice (ppm)	51	2.0%	0.8%	0.01	64	1.2%	0.7%	0.12
Turmeric (ppm)	63	0.8%	0.4%	0.05	65	0.6%	0.4%	0.11`
Soil (ppm)	67	1.8%	1.0%	0.07	63	-0.4%	1.4%	0.78

For Proximal group children each 10% increase in lead concentration in dust, all spices, other spices and turmeric was associated with a significant increase in BLL. For Distal children only increased dust lead was associated with a significant increase in BLL.

In principle component analysis, the first principal component resulted in weights that were 0.00 for turmeric and -0.13 for average of other spice levels (excluding turmeric) and weights of 0.70 for dust, 0.38 for paint and 0.59 for soil. The second component had 0.74 for turmeric and 0.66 for other spices while the weights for dust, paint and soil were all ≤ 0.11 indicating spices were independent of other environmental exposures.

### Multivariate analyses

**Fig 2:**
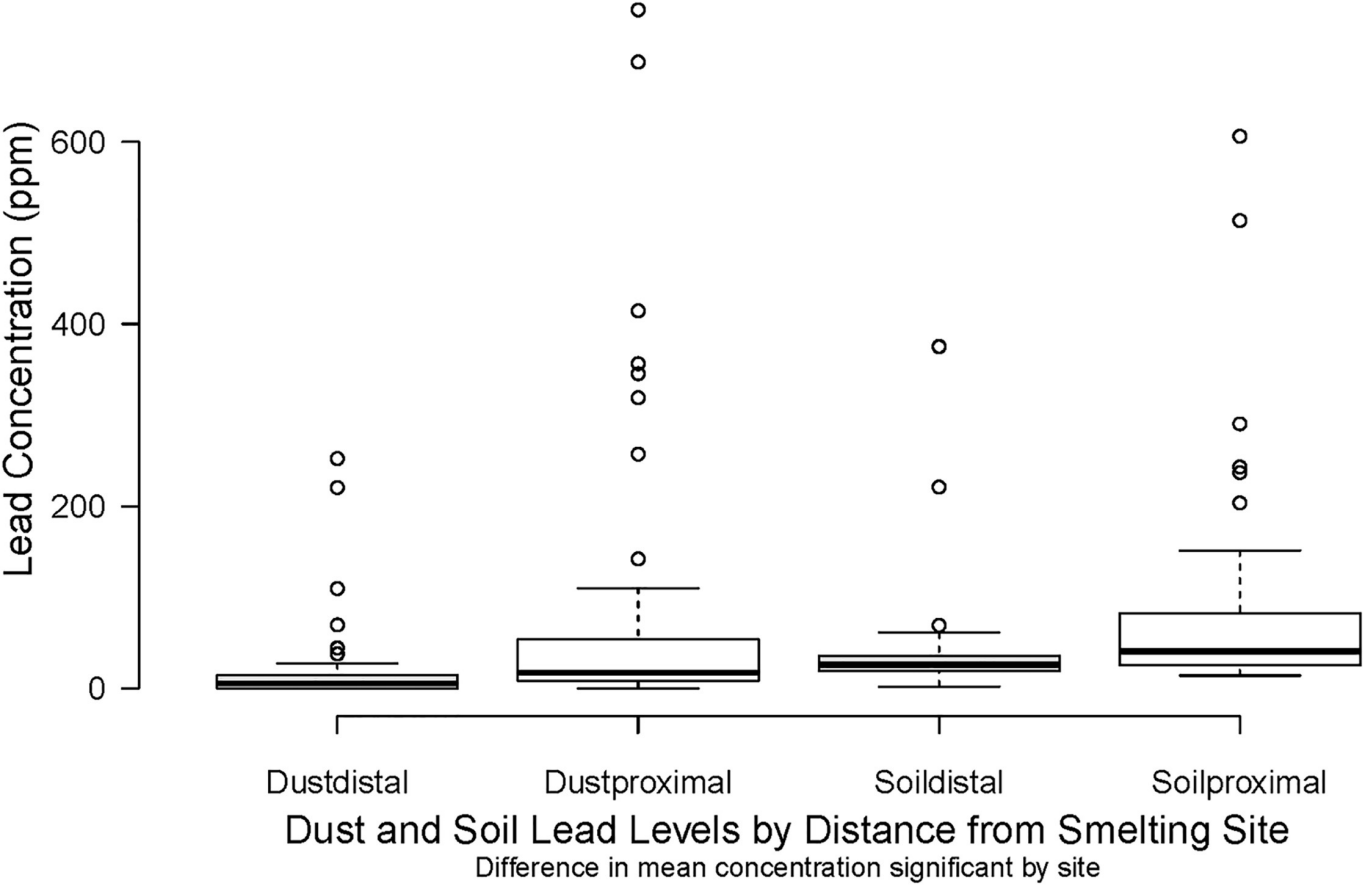
Dust and soil samples.

In bivariate models, the GM BLLs for all children was significantly higher in third vs. first tercile for dust wipe, other spice and turmeric lead levels. GM BLL ratios are also higher by tercile for dust wipe, (GM 9.5 vs. 10.6 μg/dL (p = 0.03)) turmeric (GM 8.5 vs. 14.4 μg/dL (p<0.01)) and other spice (GM 7.5 vs 12.3 μg/dL (p<0.01)). The difference in BLL by soil lead tercile is not significant. (Table 9). Stratified by group assignment, the GM BLL for Proximal children to the third vs. first tercile are significantly higher for other spice (GM 7 vs. 13.7 μg/dL (p = 0.03)) but not for soil, dust or turmeric. For Distal children GM BLL in the third vs. first tercile are significantly different for lead levels in dust wipes (GM 10.1 vs. 17.5 μg/dL (p = 0.02)) (Table 10 and Figs 1 & 2). For both groups the ratio of GM trends to higher BLL for higher lead concentrations in all environmental samples but this difference is not significant (Table 10).

**Table 9:** Bivariate analysis of BLL and environmental lead levels in terciles for all children. Patna Bihar, February 2020.

Environmental	Combined (n = 135)			
Sample Type	N	Geometric Mean BLL	Ratio of Geometric means	P value
**Dust Wipe** (μg/ft^2^)				0.10
1 = 0.01 to 3.94	45	9.53	Ref	
2 = 4.21 to 17.88	45	12.02	1.26	0.17
2317.97 to 745.06	45	10.62	1.43	0.03
**Other Spice**				0.002
1 = 2.12 to 9.56	38	7.54	Ref	
2 = 10.00 to 46.56	39	12.28	1.63	0.01
3 = 47.00 to 829.00	38	14.23	1.89	< 0.01
**Turmeric**				0.01
1 = 3.54 to 37.00	42	8.51	Ref	
2 = 41.00 to 816.00	43	11.45	1.34	0.08
3 = 825.00 to 5773.00	43	14.36	1.69	< 0.01
**Soil (ppm)**				0.61
1 = 8.49 to 25.00	46	10.43	Ref	
2 = 25.50 to 41.00	41	12.14	1.16	0.39
3 = 42.00 to 3755.00	43	12.05	1.16	0.40

Comparing the GM BLL for all children to lead concentrations in environmental media, BLLs are significantly higher in third vs. first tercile for dust wipe, other spice and turmeric lead levels. GM BLL ratios are also higher by tercile for dust wipe, other spice. The difference in BLL by soil lead tercile is not significant.

**Table 10:** Bivariate analysis of BLL and environmental lead levels in terciles stratified by distance to ULAB activities. Patna Bihar, February 2020.

Environmental	Proximal (n = 67)				Distal (n = 68)			
Sample Type Terciles	n	Geometric Mean BLL	Ratio of Geometric means	Within group P value	n	Geometric Mean BLL	Ratio of Geometric means	Within group P value
**Dust Wipe** (μg/ft^2^)				0.27				0.03
0 = 0.01 to 3.94	13	8.0	ref		32	10.1	ref	
1 = 4.21 to 17.88	23	9.4	1.2	0.59	22	15.6	1.53	0.03
2 = 17.97 to 745.06	31	12.2	1.5	0.14	14	17.5	1.70	0.02
**Other Spice**				0.07				0.212
0 = 2.12 to 9.56	27	7.0	ref		11	9.1	ref	
1 = 10.00 to 46.56	12	10.5	1.49	0.18	27	13.2	1.45	0.16
2 = 47.00 to 829.00	12	13.7	1.96	0.03	26	14.5	1.60	0.08
**Turmeric**				0.21				
0 = 3.54 to 37.00	29	8.1	ref		13	9.5	ref	
1 = 41.00 to 816.00	17	10.0	1.32	0.25	26	12.5	1.32	0.25
2 = 825.00 to 5773.00	17	14.0	1.54	0.08	26	14.6	1.54	0.08
**Soil (ppm)**				0.47				0.20
0 = 8.49 to 25.00	16	9.6	ref		30	10.9	ref	
1 = 25.50 to 41.00	18	8.7	0.91	0.74	23	15.8	1.45	0.08
2 = 42.00 to 3755.00	33	11.6	1.22	0.46	10	13.7	1.25	0.42

Comparing the GM BLL for Proximal children to lead concentrations in environmental media, BLL in the third vs. first tercile are significantly higher for other spice. For Distal children BLL in the third vs. first tercile are significantly different for lead levels in dust wipe. For both groups the ratio of GM trends to higher BLL for higher lead concentrations but this difference is not significant. The difference in BLL by soil lead tercile is not significant.

Eleven social and environmental variables significant at p≤ 0.10 were used in multivariable linear regression analysis, lead concentration in turmeric, other spice, and dust-wipes as well as number of adults in the household, living on the second floor or higher, type of floor, number of rooms in the house, ULAB materials in the house, lead-related occupation by family members, source of spices and group assignment. (Proximal vs. Distal) These were entered into the final model. For this Combined group, categorical age was forced into the model. The final model included group assignment, age, number of rooms in the house and lead concentration in turmeric and dust wipes. The model explains 19% of the variation in BLL (Table 11).

**Table 11:** Backward models of BLLs and variables significant at p <0.10, categorical age forced into the model and with R-Squared all children Patna Bihar, 2020.

	n = 107, R^2^ = 28%		n = 123, R^2^ = 19%	
	Full Model		Final Model	
Category	Ratio	p	Ratio	p
**Proximal (ref = Distal)**	0.83	0.35	0.73	0.04
**Age**				
8–23 months (ref)				
24–47 months	1.07	0.76	1.06	0.76
48–71 months	1.32	0.23	1.19	0.37
**Number of adults in household (ref = >4)**	1.18	0.35		
**Spice purchase source**				
Branded (ref)				
Loose Spice only	1.25	0.35		
Other Combination or None	1.18	0.64		
**Level of household in building**				
Ground (ref)				
First Floor	1.06	0.83		
Second/Third Floor	0.72	0.24		
**Number of rooms in house (ref = >3)**	1.31	0.19	1.49	0.01
**Turmeric log (ppm)**				
3.54 to 37.00 (ref)				
41.00 to 816.00	1.25	0.29	1.34	0.08
825.00 to 5773.00	1.77	0.01	1.87	0.0003
**Other spice log (ppm)**				
2.12 to 9.56 (ref)				
10.00 to 46.56	1.16	0.47		
47.00 to 829.00	1.33	0.21		
**Dust wipe log (μg/ft** ^**2**^ **)**				
0.01 to 3.94 (ref)				
4.21 to 17.88	1.33	0.16	1.42	0.04
17.97 to 745.06	1.26	0.30	1.61	0.01
**Soil log (ppm)**				
8.49 to 25.00 (ref)				
25.50 to 41.00	1.17	0.43		
42.00 to 3755.00	1.12	0.58		

In the parsimonious model controlling for variables significant at p<0.10 in bivariate analyses, spices, turmeric, and number of rooms in the house were significant. Controlling for these factors the Proximal group had a significantly lower BLL. The model explains 19% of the variability in BLLs.

For the Proximal group, the final parsimonious model variables were lead concentration in turmeric, other spices, number of adults in the household and age explaining 23% of the variance in BLLs (Table 12). The Distal group final model contained lead concentration in soil and turmeric and spice purchase source, the number of rooms in a household and age; explaining 45% of the variance in BLLs. (Table 13).

**Table 12:** Backward models BLLs and variables significant at p <0.10, categorical age forced into the model with R-Squared Proximal group Patna Bihar, 2020.

	n = 50, R^2^ = 32%		n = 50, R^2^ = 23%	
	Full Model		Final Model	
Category	Ratio	p-value	Ratio	p-value
**Age**				
8–23 months (ref)				
24–47 months	0.83	0.64	0.89	0.73
48–71 months	0.94	0.87	1.12	0.74
**Number of adults in household (ref = >4)**	1.33	0.37	1.55	0.10
**Spice purchase source**				
Branded (national) only (ref)				
Loose (local) only	1.06	0.87		
Other Combination or None	0.58	0.43		
**Level of household in building**				
Ground (ref)				
First Floor	1.05	0.92		
Second/Third Floor	0.85	0.75		
**Number of rooms in house (ref = >3)**	1.16	0.74		
**Turmeric log (ppm)**				
3.54 to 37.00 (ref)				
41.00 to 816.00	1.37	0.40	1.27	0.46
825.00 to 5773.00	2.07	0.09	1.70	0.10
**Other spice log (ppm)**				
2.12 to 9.56				
10.00 to 46.56	1.13	0.75	1.35	0.36
47.00 to 829.00	1.51	0.33	1.87	0.06
**Dust wipe log (μg/ft** ^**2**^ **)**				
0.01 to 3.94				
4.21 to 17.88	1.56	0.27		
17.97 to 745.06	1.12	0.79		
**Soil log (ppm)**				
8.49 to 25.00				
25.50 to 41.00	0.94	0.87		
42.00 to 3755.00	1.39	0.39		

In the parsimonious model controlling for variables significant at p<0.10 in bivariate analyses, only the categorical other spice remains significant. The model explains 23% of the variability in BLLs.

**Table 13:** Backward models Of BLLs and variables significant at p <0.10, categorical age forced into the model with R-Squared Distal group Patna Bihar, 2020.

	n = 57, R^2^ = 49%		n = 60, R^2^ = 45%	
	Full Model		Final Model	
Category	Ratio	P	Ratio	p
**Age**				
8–23 months (ref)				
24–47 months	1.54	0.13	1.55	0.05
48–71 months	2.25	0.01	2.25	0.002
**Number of adults in household (ref = >4)**	1.19	0.45		
**Spice purchase source**				
Branded national only (ref)				
Loose spice only	3.42	0.01	3.06	0.004
Other combination or none	4.74	0.004	4.77	0.001
**Level of household in building**				
Ground (ref)				
First Floor	1.20	0.59		
Second/Third Floor	0.64	0.32		
**Number of rooms in house (ref = >3)**	1.44	0.12	1.60	0.01
**Turmeric log (ppm)**				
3.54 to 37.00 (ref)				
41.00 to 816.00	1.02	0.95	1.01	0.96
825.00 to 5773.00	1.65	0.06	1.56	0.04
**Other spice log (ppm)**				
2.12 to 9.56 (ref)				
10.00 to 46.56	0.92	0.76		
47.00 to 829.00	1.01	0.98		
**Dust wipe log (μg/ft** ^**2**^ **)**				
0.01 to 3.94 (ref)				
4.21 to 17.88	1.19	0.47		
17.97 to 745.06	1.30	0.31		
**Soil log (ppm)**				
8.49 to 25.00 (ref)				
25.50 to 41.00	1.66	0.04	1.85	0.004
42.00 to 3755.00	1.06	0.83	1.17	0.54

In the parsimonious model controlling for variables significant at p<0.10 in bivariate analyses, spices, turmeric, number of rooms in the house and soil lead concentration remain significant. The model explains 45% of the variability in BLLs.

## Discussion

This study found that high BLLs in children in Patna were common. Of the 135 children enrolled, 87% had BLLs ≥5 μg/dL and 68% had levels ≥10 μg/dL. Overall, the average BLL was 14.9 μg/dL, which was higher than the mean BLL of 6.9 μg/dL estimated through a recent meta-analysis of relevant studies in India.^10^ In comparison, children (3–12 years) living around another ULAB site, Karmalichak, Patna, had higher average BLLs (24.4 μg/dL) in 2019 [14]. BLLs in this range are known to cause behavioral and intellectual disabilities that prevent children from achieving in school and increase violent and other criminal behavior. In impoverished areas such as Patna this can have a substantial impact at the community level when these deficits combined with other social determinants of well-being affect the community’s economic success [31, 32].

The hazards of lead in house paint and kohl are well understood. In this study we found few lead paint hazards in housing and less than 1/3 of children used kohl. However, we found that children in both groups were frequently exposed to a variety of other lead sources. There were significant differences in the lead exposure sources, most notably dust and spices, between the groups which were independent of each other and associated with elevated BLLs. More than three times the number of Proximal children had indoor dust concentrations above the EPA threshold compared to Distal children. This may be explained by lead in dust becoming entrained in a poorly ventilated housing and recirculated when floors are swept, as has been reported in other areas with high ambient lead contamination [33].

For Distal group children, turmeric lead levels were significantly higher compared to those for Proximal children. Compared to the concentration of lead in turmeric identified in Bangladesh and among South Asian populations in the United States, the levels recorded in Patna were extremely high, 2–3 orders of magnitude higher than the Food Safety and Standards Authority of India (FSSAI) acceptable level of < 10 ppm [34, 35]. Consistent with other studies, the higher turmeric lead levels among the Distal group are likely due to their more frequent use of loose, local, unpackaged turmeric compared to the Proximal group. Contaminated spices may also underlie the statistically significant association between categorical age and BLL for the Distal group, not seen in the Proximal group if spice consumption increases with age.

A study conducted in Bangladesh found that loose turmeric is more commonly adulterated with lead chromate pigment. The study further revealed that lead chromate was being added during the polishing stage to enhance the yellow color of turmeric roots [28]. Although the reason for adding lead chromate to turmeric in Patna may be for color as it was in Bangladesh, the molar ratio of lead to chromium was also indicative of lead chromate among samples of chili and coriander in this study. Chili and coriander do not share turmeric’s vibrant yellow hue, so the evidence of lead chromate (yellow) and not lead oxide (red) pigment addition is surprising. Given that chili and coriander had far lower average and maximum lead concentrations than turmeric in this study, one possible explanation may be that chili and coriander get contaminated by residual lead chromate from shared grinding equipment or storage bins.

Unexpectedly, given the findings of previous investigations where extraordinarily high soil lead levels were identified, we did not find high soil lead levels in the areas where ULAB activities had been conducted. This may be because informal ULAB recycling is a somewhat transient activity that responds to market demands as well as the availability of the batteries, but even in legacy sites with no recently released lead, soil lead levels remain high for years to decades and do not decline without active remediation [36]. The areas in this study were flooded with up to 8 feet of water in August 2019, 6 months before our study commenced which could have diluted soil lead levels, similar to what has happened elsewhere after flooding events. In 2005, two major hurricanes engulfed the city of New Orleans, USA, flooding the city and washing tons of soil and debris into the Gulf of Mexico. Prior to the floods, average soil lead levels were ≥1000 ppm in several neighborhoods. Ten years after the floods, soil lead levels were dramatically lower with none >600 ppm and some less than 200 ppm. BLLs for the city’s children showed a similar decline with the percent of children with BLLs ≥5 μg/dL falling from about 63% to 8% in the highest risk areas [37]. Fortunately for New Orleans, the primary source of lead in these homes was lead paint and, thus recontamination is unlikely. However, this may not be the case in Patna as economic pressures combined with increasing demand for vehicles, including laudable efforts to increase the use of electric vehicles, many of which still use lead-acid batteries, and solar energy storage, will likely sustain informal battery recycling and repair an attractive source of income.

## Limitations and strengths

Our study has a number of limitations. The most notable of these is the small sample size. The reasons for this are common among community-based studies and include families not home when visited, parental refusal to participate and resource constraints [38, 39]. Our sample size restricted efforts to evaluate effect modification between environmental and demographic variables. Another limitation is that we did not expect to find high lead levels in spices, thus our study was not designed or powered to fully investigate the production, consumption patterns and quantity of spices ingested by the children. Nonetheless, this study strongly demonstrates that contaminated spices are a significant source of lead exposure for young children in Patna. Another strength of our study is its use of a modified CASPAR design. Using a randomized trial rather than convenience samples allows estimation of the distribution of BLLs in the population of interest and can assist policy maker designing remediation and prevention interventions.

## Opportunity for future research

We found lead in cookware, which is known to increase lead exposure through food, although metal concentrations based on XRF data are only weakly correlated with the amount of lead released during cooking [40]. There are currently no Indian regulatory standards for lead in cookware. Further study of cookware using more robust leaching analyses of the amount of lead released during use are needed.

High lead levels in water were only found in samples from bore holes. Contamination of these deep-water wells requires further investigation to establish the source(s) of lead (e.g., widespread groundwater/aquifer contamination or leaching of lead from lead pipes and fittings).

Study of the supply chain for local turmeric and other loose spices is needed to determine where the contamination occurs and how to eliminate lead from the process. Further study could also improve our understanding of consumption patterns especially for young children. In the meantime, families should be cautioned against using these spices in food, especially foods consumed by children and pregnant women.

Finally, given the many and varied lead sources in the environments of children in Patna, further study that enrolls a larger number of children and provides more detail on the behavioral and nutritional status of children at different ages, including dietary intake would provide additional information for policy and decision makers.

## Conclusions

Children in Patna, Bihar, India are exposed to multiple sources of lead, with lead levels in house dust and loose, locally sourced spices being the most likely to increase blood lead levels. This indicates that children can absorb lead from any source Thus, a holistic approach to identify and mitigate all sources of lead exposure is needed. For both groups of children, a 10% increase in the lead content of dust, turmeric, or other spices each resulted in a 0.7–2.0% increase in BLLs.

We found lower soil lead levels in this study compared to previous studies in India and elsewhere in areas where ULAB activities are conducted. This may be the result of the ‘natural’ clean-up of contaminated soils due to severe flooding in areas where ULAB have been recycled. Nothing in this study should be interpreted to indicate that ULAB activities do not contaminate soil and dust in adjacent communities.

Given the prevalence of BLLs ≥ 5 μg/dL, a level recognized by the World Health Organization as elevated, BLL surveillance of children should be integrated into routine pediatric care and vaccination programs. Efforts to increase awareness and to clean up interior dust and soil lead hazards should be implemented in homes with young children living in Patna. Clean up measures are well understood, do not require advanced technology, and have been demonstrated to reduce BLLs in children over time. Increased monitoring and strengthened oversight of lead content in consumer products, foodstuff and spices is also needed to ensure consumer safety.
